# Numerical Prediction and Experimental Validation of Deposited Filaments in Direct Ink Writing: Deposition Status and Profile Dimension

**DOI:** 10.3390/polym17050573

**Published:** 2025-02-21

**Authors:** Yongqiang Tu, Haoran Zhang, Xue Shi, Jianyu Fan, Baohua Bao, Gang Lu, Fuwei Han, Hao Wu, Alaa Hassan

**Affiliations:** 1 College of Marine Equipment and Mechanical Engineering, Jimei University, Xiamen 361021, China; 202361000149@jmu.edu.cn (Y.T.); 202412855070@jmu.edu.cn (H.Z.); luetao696292@163.com (B.B.); 202321367045@jmu.edu.cn (G.L.); 202321363062@jmu.edu.cn (F.H.); 18300939269@163.com (H.W.); 2 China Three Gorges New Energy Corp, Beijing 101199, China; shi_xue@ctg.com.cn; 3 Équipe de Recherche sur les Processus Innovatifs (ERPI), Université de Lorraine, F-54000 Nancy, France; alaa.hassan@univ-lorraine.fr

**Keywords:** deposited filaments, direct ink writing, numerical simulation, deposition status, profile dimension

## Abstract

The deposition status and profile dimension of deposited filaments have an impact on the quality of the printed parts fabricated by direct ink writing (DIW). Previous works often failed to realize the full quantitative characterizations of the detailed influence of the process parameters on the deposition status and profile dimension. Herein, we predict and analyze the deposition status and profile dimension by proposing an improved three-dimensional (3D) numerical model. The prediction accuracy of the proposed numerical model is verified through filament deposition experiments. The maximum relative errors of width and height between the experimental and simulation results of cross-sections are 10.13% and 7.37%, respectively. The effect of process parameters on the deposition status and profile dimension has been quantified. Critical process parameters are identified as the dimensionless nozzle velocity (V*) and the dimensionless height (H*). Three deposition statuses named over-deposition, pressed deposition and freeform deposition are characterized depending on the combination of V* and H*. The current work demonstrates an effective approach for the prediction of the deposition status and profile dimension of the deposited filaments along with the investigation of the effects of process parameters in DIW based on numerical simulations.

## 1. Introduction

Direct ink writing (DIW), which belongs to the material extrusion additive manufacturing (MEAM) techniques [1], is one of the most promising and flexible types of AM methods with the primary strength in the diversity of available materials including polymers [2,3,4]. The process principle of DIW is preparing the material in paste or slurry type as an ink with shear-thinning and viscoelastic behavior first [5], then extruding the prepared ink from a nozzle into freeform continuous filaments with mechanical force (piston or screw drive) or pneumatic force [6], and finally depositing the freeform extruded filaments onto a substrate in a layer-by-layer fashion by moving the nozzle [7].

The quality of the DIW 3D printed parts depends on the deposition status and profile dimension of deposited filaments [8]. Material properties [9] (including density, surface tension coefficient, static contact angle and rheological properties) and process parameters [10] affect the deposited filaments. In order to fully understand the process and predict the deposited filaments, many efforts have been made in deposited filament modeling and prediction for MEAM to find the relationship between process parameters with profile dimensions [11,12,13]. The modeling methods for the relationship between process parameters with profile dimensions mainly include experimental methods [14], analytical methods [15] and numerical simulation methods [16]. Experimental methods present a regression model between input process parameter factors and experimental profile dimensions results [17], but these methods are complex and material/time-consuming considering the different material properties of variable inks and the large number of printing variables involved in DIW [18]. Analytical methods offer a model for the relationship between process parameters with profile dimensions based on the mathematical description and derivation of the physical process, but the prediction accuracy of these methods is restricted because the cross-sectional profile needs to be simplified as a constant shape such as half-ellipse [19], circular [20], rectangle, oblong and ellipse [21] in analytical modeling. However, the cross-sectional profile of deposited filaments will change under different conditions of inks and process parameters [22]. Compared with experimental and analytical methods, numerical simulation methods can be applied as more suitable methods to predict deposited filaments in DIW to fully find the relationship between process parameters and profile dimensions [23,24,25].

In recent years, several researchers have established numerical prediction models for deposited filaments in MEAM to find the relationship between process parameters and profile dimensions. Comminal et al. [26] established a numerical model of deposited filaments in MEAM. This work quantified the effect of the gap distance and the velocity ratio on the size and the shape of the strand. The cross-section of the strand ranged from being almost cylindrical (for a fast printing and with a large gap) to a flat cuboid with rounded edges (for a slow printing and with a small gap). But this work lacked experimental validation. Serdeczny et al. [27] used an experimental procedure to validate the numerical model and capture the changes in the strand morphology for the different processing conditions. It was found that the cross-section of the strand could vary from being almost circular to an elongated rectangular shape with rounded edges depending on the processing conditions. Göhl et al. [28] used a computational fluid dynamics (CFD) simulation tool to study the effect of printing parameters on deposited filaments in bio-printing. The results showed that simulation was useful for determining the impact on line resolution and viscoelastic stresses when changing printing parameters such as printing speed and nozzle height. Gosset et al. [29] presented a numerical study of the deposition of a single strand of poly-lactic acid (PLA) by MEAM via a CFD model. It was found that the final quality of the pieces was strongly linked to the shape, size and surface finish of the strands deposited successively, which themselves depended on the printing parameters and extruded material properties. Rashid et al. [30] established a numerical model for the thermomechanical performance of MEAM. In this work, two crucial process parameters (i.e., extrusion temperature and layer resolution) were varied while keeping the other process parameters, part geometry, and material properties constant to optimize the process parameters. Balani et al. [31] studied the influence of process parameters on material deposition and the rheological properties to improve the quality of the profile dimensions for deposited filaments in MEAM using numerical methods. Although the previous works found that filament deposition is significantly influenced by process parameters, they often failed to realize full quantitative characterizations of the detailed influence of the process parameters on the deposition status and profile dimension of deposited filaments. Meanwhile, few studies have been carried out on ink material’s properties and process parameter settings. Thus, it is crucial and desirable to fully study and predict the deposition status and profile dimension of the deposited filaments in DIW processes based on numerical prediction.

In this work, an improved 3D numerical prediction model of deposited filaments in DIW is established with the volume of fluid (VOF) method to predict the deposition status and profile dimension of deposited filaments. A commercially available microcrystalline wax (MW)-based ink is selected as an ink reference and filament deposition experiments are conducted using the ink to verify the proposed model. Using the proposed numerical model, the deposition status is divided into three statuses and the profile dimension under each status is predicted. The effect of process parameters including the dimensionless nozzle velocity and the dimensionless height on deposition status and profile dimension has been quantified using numerical simulations for the first time. The work demonstrates a material-saving and effective approach for the prediction of deposition status and profile dimension of the deposited filaments and the investigation of the effects of process parameters in DIW with computer-based numerical simulations.

## 2. Materials and Methods

### 2.1. Numerical Model Development

#### 2.1.1. Objective Description

**Figure 1:**
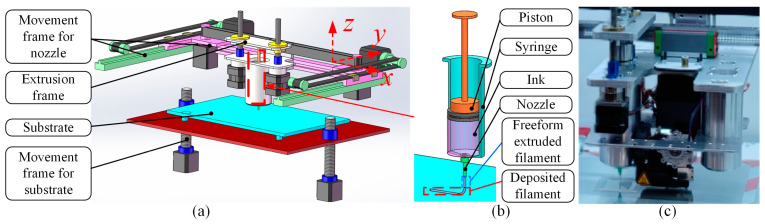
Description of the DIW process and deposited filament: (**a**) sketch of DIW process; (**b**) sketch of deposited filament; (**c**) picture of DIW process.

As illustrated in Figure 1, the critical mechanical components of a DIW 3D printer include movement frame for nozzle, extrusion frame, substrate, movement frame for the substrate, piston, syringe and nozzle. In the DIW process, ink is filled in the syringe and the piston is driven by the extrusion frame at a constant velocity to extrude the ink from a nozzle into continuous freeform extruded filaments. Then, the movement frame for the nozzle guides the nozzle into moving towards the *x*-*y* plane at a constant distance between the nozzle bottom and substrate, which is controlled by the movement frame for the substrate to deposit freeform extruded filaments on the substrate as deposited filaments. The numerical model of freeform extruded filaments has been established in our previous work [32]. However, the previous model is a simplified two-dimensional simulation that cannot predict the deposition status and profile dimension of the deposited filaments. Meanwhile, 3D numerical models of deposited filaments in previous works ignore some important material properties such as the surface tension coefficient and static contact angle, which limit the prediction accuracy. The objective of this work is to focus on the 3D numerical modeling of deposited filaments on the substrate considering the overall material properties to predict their deposition status and profile dimension, and to investigate the effect of process parameters on the deposition status and profile dimension.

### 2.1.2. Numerical Method and Governing Equations

The 3D numerical modeling for deposited filaments is a two-phase flow simulation to capture the interface between the ink and air. Numerical methods for two-phase flow problems include the level set method [33], phase field method [34], lattice Boltzmann method [35], direct interface tracking method [36] and VOF method [37]. Among these numerical methods, the VOF method has been widely used in numerical simulations of filaments in MEAM due to its simplicity, robustness and straightforward implementation [38]. However, previous simulations for deposition filaments using VOF ignored surface tension and static contact angles. Hence, an improved 3D numerical model achieved using the VOF method and by considering the overall ink material properties is required, which is the focus of the current study.

In the 3D numerical simulation modeling for deposited filaments using VOF, the ink and air are treated as a single continuum and the phase fraction α is defined to distinguish the interface between the two-phase fluid as follows.

(1) $$\alpha =\frac{V_{i}}{V_{m}}$$

where α is the phase fraction for a mesh; $V_{i}$ is the volume of the ink in a mesh; $V_{m}$ is the total volume of a mesh. Values of α are between 0 and 1 and α = 0.5 is defined as the cutoff value to capture the sharp boundary of the interface between the ink and air.

Fluid properties of the single continuum are weighted with the phase fraction as follows.

(2) $$\rho_{s}=\alpha \rho +\left(1-\alpha \right)\rho_{a},$$

(3) $$\mu_{s}=\alpha \mu +\left(1-\alpha \right)\mu_{a},$$

where $\rho_{s}$ and $\mu_{s}$ are the density and viscosity of the single continuum; ρ and $\rho_{a}$ are the densities of the ink and air, respectively; μ and $\mu_{a}$ are the viscosities of the ink and air, respectively.

The ink has shear-thinning behavior and its viscosity μ is expressed using the Hershel–Bulkley model as follows [39]:

(4) $$\mu =\min\left(\mu_{0},\tau_{0}/\dot{\gamma }+K{\dot{\gamma }}^{n-1}\right),$$

where $\dot{\gamma }$ (unit: $s^{-1}$) is the shear rate; $\mu_{0}$ (unit: Pa⋅s), $\tau_{0}$ (unit: Pa), K (unit: $Pa\cdot s^{n}$) and n (unit: dimensionless) are rheological properties defined as the limiting dynamic viscosity, yield stress, consistency index, flow index for inks. If stress < $\tau_{0}$, the ink behaves as a rigid solid, otherwise it behaves as a fluid. The ink is shear-thinning when the value of n is less than 1. The values of $\mu_{0}$, $\tau_{0}$, K and n are determined through rheological experiments.

The following assumptions about the filament deposition simulation are formulated:

- The deposition process is considered isothermal as the process is conducted at room temperature;
- The material is considered incompressible with the constant ρ;
- The ink adheres to the surfaces of the nozzle without slip and the static contact angle between the ink and the substrate is considered;
- The filament flow is laminar.

Based on these assumptions, the governing equations of 3D shape for deposited filaments include continuity equation, momentum equilibrium equation and phase fraction equation. Each equation of the 3D deposited filaments modeling is written as follows.

Continuity equation:

(5) ∇⋅U=0,

where U is velocity field of the fluid.

Momentum equilibrium equation:

(6) $$\frac{\partial \rho_{s}U}{\partial t}+\nabla \cdot \left(\rho_{s}UU\right)=-\nabla p+\nabla \cdot \left(\rho_{s}\mu_{s}\nabla U\right)+\rho_{s}g+F_{\sigma },$$

where p is the pressure in the fluid. g is the gravitational acceleration vector; $F_{\sigma }$ is surface tension. $F_{\sigma }$ is defined as follows:

(7) $$F_{\sigma }=\sigma \kappa \nabla \alpha ,$$

where σ is the surface tension coefficient; κ is the surface curvature, which depends on the shape of deposited filaments. κ is computed from local gradients in the surface normal compared to the interface as follows:

(8) $$\kappa =\frac{1}{\left|n\right|}\left(\left(\frac{n}{\left|n\right|}\cdot \nabla \right)\left|n\right|-\nabla \cdot n\right),$$

where n=∇α is the normal vector to the ink surface. Wall adhesion between the deposited filaments and substrate is included in the model through the static contact angle as follows:

(9) $$\hat{n}={\hat{n}}_{w}\cos\theta +{\hat{t}}_{w}\sin\theta ,$$

where $\hat{n}=n/\left|n\right|$ is the unit vector normal to the ink surface. ${\hat{n}}_{w}$ and ${\hat{t}}_{w}$ represent the unit vector normal and tangent to the wall, respectively. θ is the static contact angle between the ink and substrate.

Phase fraction equation:

(10) $$\frac{\partial \alpha }{\partial t}+\nabla \cdot \left(\alpha U\right)+\nabla \cdot \left(\alpha \left(1-\alpha \right)U_{r}\right)=0,$$

where $U_{r}$ is the velocity vector compressing the two-phase free surface, which represents the velocity difference between two-phase fluids and can be calculated as follows:

(11) $$U_{r}=\min\left(c\left|U\right|,\max\left|U\right|\right)\frac{\nabla \alpha }{\left|\nabla \alpha \right|},$$

where c is a controllable compression factor and it is selected as c = 1 in the numerical simulation.

### 2.1.3. Numerical Modeling

**Figure 2:**
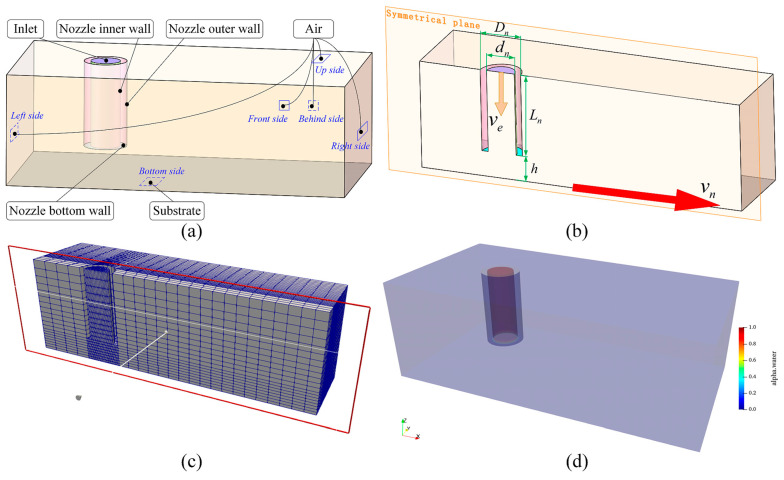
Numerical modeling of 3D profile of deposited filaments: (**a**) 3D geometrical model; (**b**) critical parameters in the model; (**c**) generated meshes; (**d**) initial condition of phase fraction.

Numerical modeling for the 3D profile of deposited filaments is established based on the use of free and open-source CFD software OpenFOAM v1912 [40] as shown in Figure 2. Because the ink extrusion and deposition process are considered as an isothermal, incompressible and laminar flow, the average velocity on each cross-section in the nozzle is the same and it can be expressed as follows using the conservation of mass.

(12) $$v_{e}={\left(D_{p}/d_{n}\right)}^{2}v_{p},$$

where $v_{e}$ is the average velocity of ink on each cross-section in the nozzle. $D_{p}$ is the piston diameter. $d_{n}$ is the inner diameter of nozzle. $v_{p}$ is the piston velocity.

According to Equation (12), the flow situation in the syringe has no effect on $v_{e}$ Thus, the 3D geometrical model is simplified as a combination of the nozzle, substrate and air by neglecting the syringe to improve computational efficiency as shown in Figure 2a. As presented in Figure 2b, the critical parameters in the model include the inner diameter of nozzle $d_{n}$, the outer diameter of nozzle $D_{n}$, the average velocity of ink in nozzle $v_{e}$, the nozzle length $L_{n}$, the distance between nozzle bottom and substrate h, and the nozzle velocity $v_{n}$. In this study, the values of $d_{n}$ and $D_{n}$ are set as 0.84 mm and 1.22 mm, which match the actual dimensions of the DIW 3D printer in experimental verification, respectively. $v_{e}$ is calculated using Equation (12) where the value of $D_{p}$ is set as 21.6 mm. As the nozzle length has no effect on $v_{e}$ and the final deposited filament, the value of $L_{n}$ is set as 2.44 mm, which is smaller than the practice nozzle length (18 mm) to reduce computational time. To avoid the model becoming a complex dynamic mesh model that needs a long computational time, the position of nozzle is fixed while the substrate is attached with a moving velocity $v_{n}$ in the boundary setting as the movements of the nozzle and substrate are relative to each other. As shown in Figure 2c, meshes are refined in the region of deposited filaments to improve calculation efficiency and accuracy.

A commercially available MW-based ink, Nivea Crème Art. No. 80104 (Beiersdorf Global AG, Hamburg, Germany), is selected as the ink reference as it is the printability reference and representative for ink preparation and process parameter selection for DIW [41]. The material properties of the ink were obtained experimentally in our previous work [42] and are listed in Table 1. The initial condition of the phase fraction is shown in Figure 2d where the red region represents the ink and blue region represents the air, meaning that the ink is filled in the nozzle at the initial time. Finally, the 3D profile of deposited filaments is obtained through VOF by capturing the sharp front of the interface where the value of α is 0.5. And the visualization of the deposited filaments is realized using the open-source, multi-platform data analysis and visualization software PareView (Version number: 5.12.0) [43].

### 2.2. Experimental Validation

**Figure 3:**
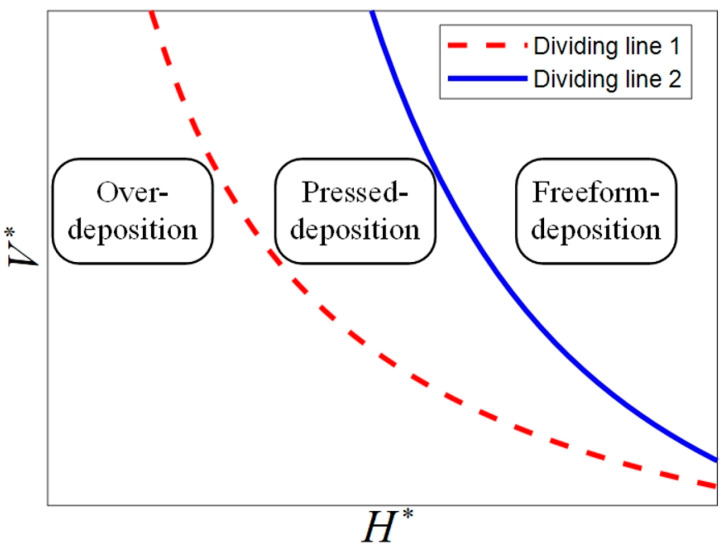
Idealized dividing of the three deposition statuses in the $H^{*}$ − $V^{*}$ figure.

Yuk and Zhao [44], Comminal et al. [26] and Athanasiadis et al. [22] have summarized that the deposition and the profile dimension of the deposited filaments in MEAM are significantly influenced by the dimensionless nozzle velocity $V^{*}$ = $v_{n}/v_{e}$ (ratio of nozzle velocity $v_{n}$ to average velocity of ink in the nozzle $v_{e}$) and the dimensionless height $H^{*}$ = $h/\beta d_{n}$ (ratio of distance between nozzle bottom and substrate h to average dimeter of freeform extruded filaments $\beta d_{n}$) where β is the die-swelling factor, which describes the post-extrusion expansion of the ink. We divide the deposition statuses into three types: over-deposition, pressed deposition and freeform deposition. In the over-deposition status, excessive ink is extruded out beyond the outer diameter of the nozzle as the nozzle bottom is too closed for the substrate or the nozzle’s displacement velocity is too slow. In the pressed-deposition status, the as-deposited filaments are pressed by the nozzle bottom but the ink stays within the region of the outer diameter of the nozzle. In the freeform deposition, there is no contact between the nozzle bottom and the as-deposited filaments as the nozzle bottom is far away from the substrate or the nozzle velocity is too fast. Using the law of conservation of mass, the idealized dividing lines for over-deposition to pressed deposition and for pressed deposition to freeform deposition are expressed in Equation (13) and Equation (14), respectively. The two dividing lines are plotted in the $H^{*}$ − $V^{*}$ figure as shown in Figure 3 where the red dotted line and blue solid line represent dividing lines determined by Equation (13) and Equation (14), respectively.

(13) $$V^{*}\leq \frac{\pi }{4\beta \gamma }\cdot \frac{1}{H^{*}},$$

(14) $$V^{*}\geq \frac{\pi }{4\beta^{2}\varepsilon }\cdot {\left(\frac{1}{H^{*}}\right)}^{2},$$

where γ is the ratio of $D_{n}$ to $d_{n}$ and ε represents $\left(\theta -\frac{\sin2\theta }{2}\right){\left(\frac{1}{1-\cos\theta }\right)}^{2}$.

As shown in Figure 3, the deposition status can be determined by $H^{*}$ and $V^{*}$ for a certain ink, so that we are able to fully evaluate the effectiveness of the numerical prediction in the deposition status and to analyze the influence of process parameters on deposition status. First, $v_{e}$ is set as the two-level process parameter, which is 6 mm/s and 9 mm/s, to investigate the influence of $v_{e}$ on the deposition status. Then, at each level of $v_{e}$, $V^{*}$ is varied from 0.4 to 1.2 at a fixed interval of 0.4 and $H^{*}$ is varied from 0.3 to 1.5 at a fixed interval of 0.3 to investigate the influence of $V^{*}$ and $H^{*}$ on deposition status.

The quantitative comparison of the profile dimension is conducted by measuring the width and height of the cross-section of the deposited filaments. A piston-driven DIW 3D printer TM-081 (Tobeca Company, Nancy, France) with the selected ink is used to conduct filament deposition experiments. In the experiments, process parameters are set first and then three 80 mm long filaments are deposited on a glass substrate and a camera (Canon LEGRIA HF R86 Noir, Canon Inc., Tokyo, Japan) is used to capture the top and side views of deposited filaments to determine the deposition status and profile dimension of the deposited filaments. The width of the deposited filaments is measured at a fixed sampling interval of 5 mm for three filaments in the top view and the height is measured in the end face for three filaments in the side view. The average and standard deviation are calculated for 45 sampling points in width measurement and 3 sampling points in height measurement. Experimental width and height are expressed as the mean ± standard deviation.

## 3. Results and Discussion

### 3.1. Experimental and Simulated Deposition Status

**Figure 4:**
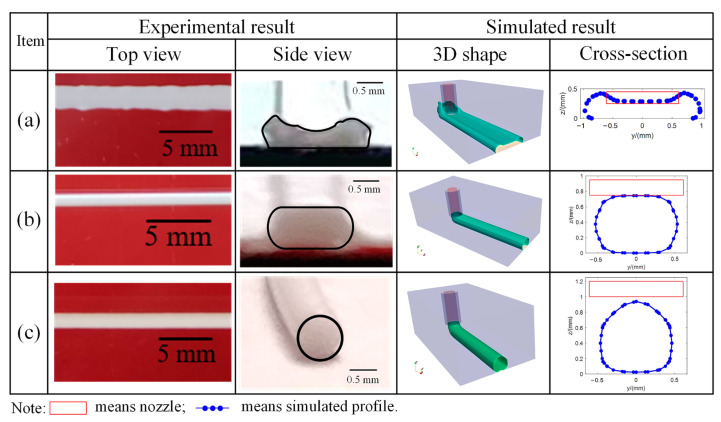
Characterization of deposition status in experimental and simulated results: (**a**) over-deposition; (**b**) pressed deposition; (**c**) freeform deposition (dynamic deposition process of three deposition statues in simulations are shown in Videos S1–S3 in Supplementary Materials).

Figure 4 presents the characterization of the three deposition statuses in the experimental results from the top view and side view and the characterization of the three deposition statuses in the simulated results from the 3D shape and cross-section, which show the good consistency of experimental and simulated results in the deposition status characterization. In the over-deposition status, the two ends of the cross-section are irregular shapes, which are higher than h. In the pressed-deposition status, the height of the cross-section is equal to h and the two ends of the as-deposited filaments possess semicircle shapes with a diameter of the same value of h. In the freeform deposition, the cross-section nearly takes a circle shape.

**Figure 5:**
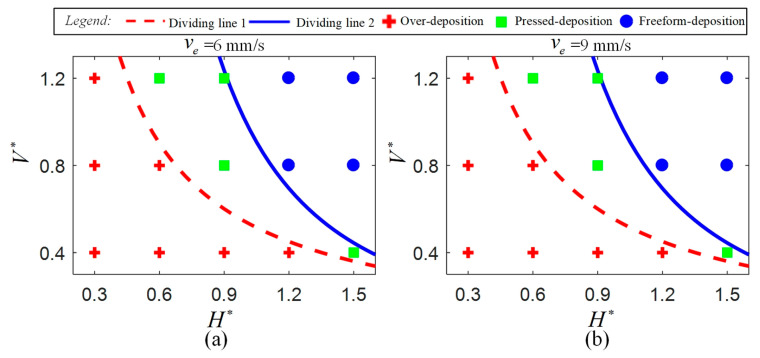
Experimental deposited status of the deposited filaments under different process parameters: (**a**) value of $v_{e}$ is set as 6 mm/s; (**b**) value of $v_{e}$ is set as 9 mm/s.

**Figure 6:**
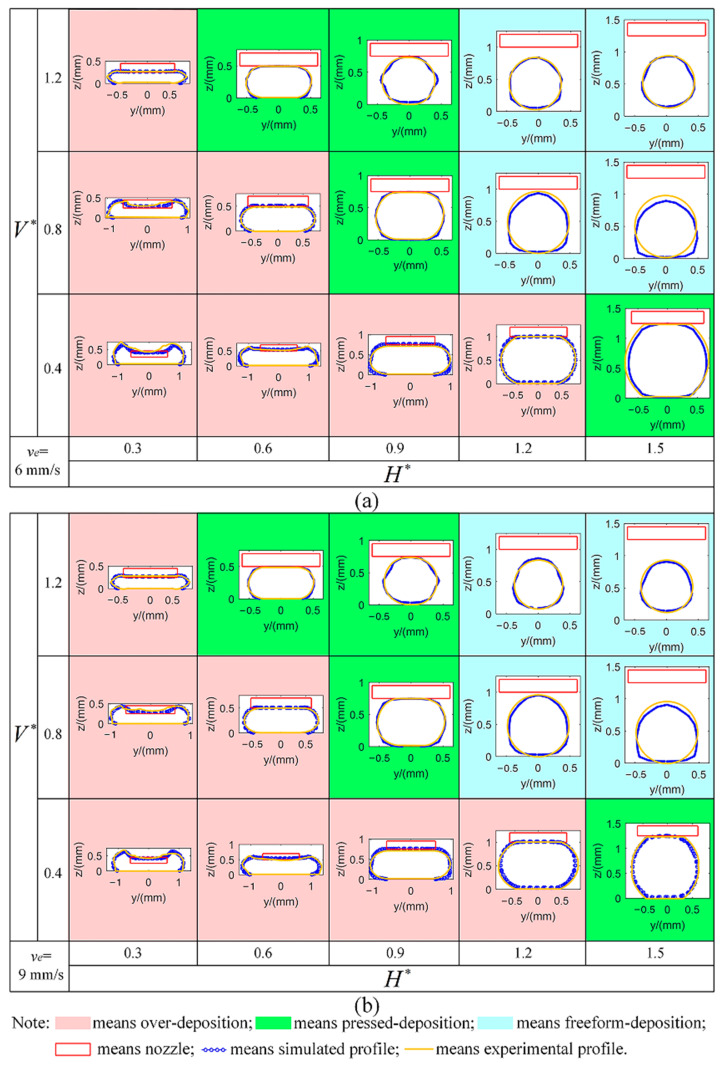
Simulated and experimental deposition statuses and profiles of the deposited filaments under different process parameters: (**a**) value of $v_{e}$ is set as 6 mm/s; (**b**) value of $v_{e}$ is set as 9 mm/s.

In order to investigate the influence of the process parameters on the deposition status, the experimental and simulated deposition statuses under different process parameters are compared. By using the symbols +, ■ and ● to represent over-deposition, pressed deposition and freeform deposition, respectively, the experimental statuses of the deposited filaments under different process parameters are summarized in Figure 5. Correspondingly, the simulated deposited filaments under different process parameters are plotted in Figure 6 by using pink, green and cyan backgrounds to represent over-deposition, pressed deposition and freeform deposition, respectively. Experimental profiles are also added using a solid gold line in Figure 6 to show the difference between the experiments and simulations. It can be found that the effectiveness of the numerical prediction for the deposited filaments is validated as the simulated deposited status dividing results are consistent with the experimental results and also consistent with the idealized results.

### 3.2. Experimental and Simulated Profile Dimension

The deposited filaments in pressed deposition and freeform deposition have regular widths and heights and can finally be deposited for 3D parts with high quality but over-deposition with irregular profiles leading to inferior quality should be avoided. Thus, prediction for width and height in pressed deposition and freeform deposition is meaningful for DIW quality evaluation and control. To verify the prediction accuracy of profile dimension for the proposed numerical model, the experimental results of width and height under different process parameter settings are measured and listed in Table 2 and Table 3 where the symbol + represented the over-deposition status. Correspondingly, the simulated width and height are listed in Table 4 and Table 5. The relative errors between experimental and the simulated width and height are calculated using the data from Table 2, Table 3, Table 4 and Table 5 and listed in Table 6 and Table 7.

**Figure 7:**
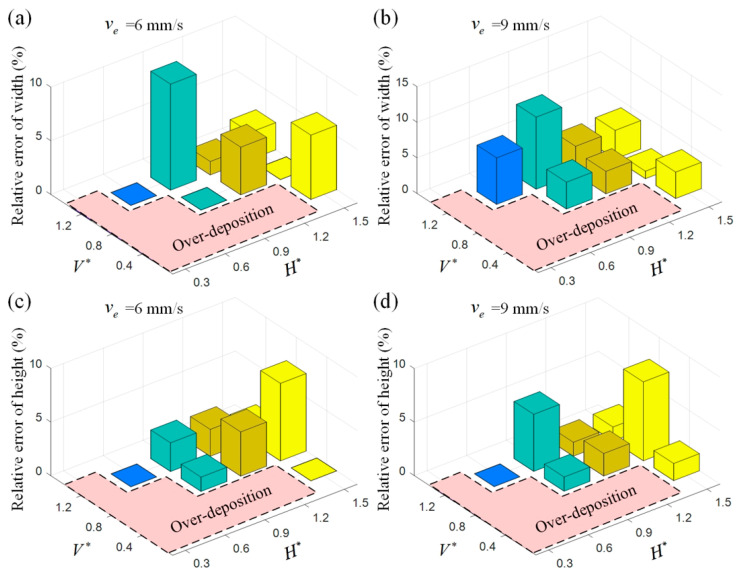
Relative error of the experimental and simulated profile dimension under different process parameters for (**a**) width when $v_{e}$ is set as 6 mm/s; (**b**) width when $v_{e}$ is set as 9 mm/s; (**c**) height when $v_{e}$ is set as 6 mm/s; (**d**) height when $v_{e}$ is set as 9 mm/s.

The accuracy of the numerical prediction for profile dimension is verified as the maximum relative errors of width and height between the experimental and simulated results are 10.13% and 7.37%, respectively, which are significantly improved as compared with the values of 20% and 28% reported in the literature [27]. As shown in Figure 7, the numerical perdition accuracy for profile dimension varies with the process parameter setting and the maximum relative error appears at the edge of the printable window. The maximum relative error of width appears at the edge of the $V^{*}$ setting ($H^{*}$ = 0.9, $V^{*}$ = 1.2) and the maximum relative error of height appears at the edge of the $H^{*}$ setting ($H^{*}$ = 1.5, $V^{*}$ = 0.8). In these cases, relatively large prediction errors for the profile dimension are generated.

### 3.3. Effects of Process Parameters

After the experimental validation for the numerical prediction of the deposited filaments, the effects of process parameters on deposited filaments are investigated based on the use of the numerical model. Figure 5 and Figure 6 and Table 4 and Table 5 show that the deposition statuses and profile dimensions are influenced by $V^{*}$ and $H^{*}$, while $v_{e}$ has no impact when $V^{*}$ and $H^{*}$ are fixed. The reason for this phenomenon is that $v_{n}$ and $v_{e}$ have the same multiple change when the value of $V^{*}$ is fixed ($V^{*}$ = $v_{n}$/$v_{e}$), which causes the effect of changes in $v_{e}$ to be offset by the synchronous changes in $v_{n}$.

**Figure 8:**
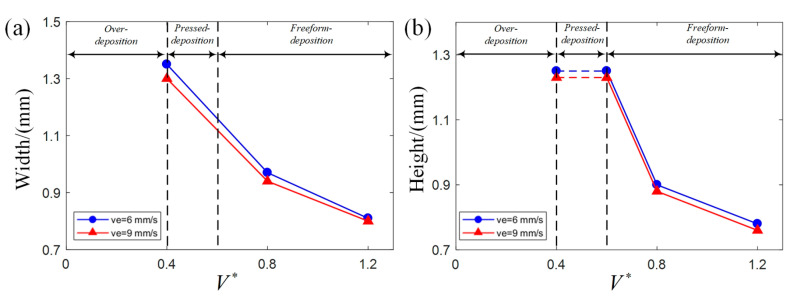
Effect of $V^{*}$ on width and height when $H^{*}$ is set as a constant value ($H^{*}$ is set as 1.5 in the two charts): (**a**) width; (**b**) height.

**Figure 9:**
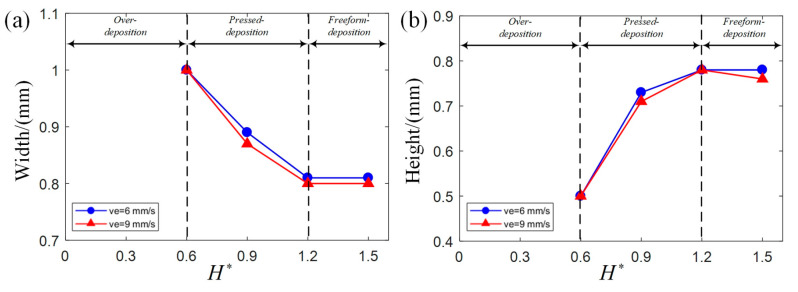
Effect of $H^{*}$ on width and height when $V^{*}$ is set as a constant value ($V^{*}$ is set as 1.2 in the two charts): (**a**) width; (**b**) height.

The combination of $V^{*}$ and $H^{*}$ influence the deposition status and profile dimension. In order to investigate the effect of $V^{*}$ and $H^{*}$ individually, one process parameter is set as a constant value while the other changes. As shown in Figure 8, to investigate the effect of $V^{*}$ individually, $H^{*}$ is set as 1.5 while $V^{*}$ changes from 0 to 1.2. As shown in Figure 9, to investigate the effect of $H^{*}$ individually, $V^{*}$ is set as 1.2 while $H^{*}$ changes from 0 to 1.5. The deposition status starts from over-deposition to pressed deposition and finally to freeform deposition when $V^{*}$ and $H^{*}$ increase as shown in Figure 8 and Figure 9. The effect of $V^{*}$ on width and height is influenced by its deposition status and is summarized as the width decreases with $V^{*}$ in pressed deposition and freeform deposition; the height remains the same with $V^{*}$ in pressed deposition but decreases with $V^{*}$ in freeform deposition. The effect of $H^{*}$ on width and height is influenced by its deposition status and is summarized as the width decreases with $H^{*}$ in pressed deposition but remains the same with $H^{*}$ in freeform deposition; height increases with $H^{*}$ in pressed deposition but remains the same with $H^{*}$ in freeform deposition.

## 4. Conclusions

The current work characterizes the deposition status and studies the profile dimension in each deposition status based on the use of numerical prediction for the first time. An improved 3D numerical model is established by using the VOF method and by considering the overall ink material properties. The improved 3D numerical model overcomes the problem of previous simplified 2D models in predicting the cross-section and the shortcoming of previous 3D numerical models in ignoring important material properties such as the surface tension coefficient and static contact angle, which limit the prediction accuracy. Critical process parameters are identified as the dimensionless nozzle velocity and the dimensionless height. Based on the experimental observations, the deposition status is characterized into three types named over-deposition, pressed deposition and freeform deposition and the profile dimension under each deposition status can be predicted using numerical simulations. The effectiveness of the numerical prediction for the deposited filaments is validated as the simulated deposited status dividing results are consistent with the experimental results and are also consistent with the idealized results. The prediction accuracy of the numerical prediction for the profile dimension is verified as the maximum relative errors of width and height between the experimental and simulated results are 10.13% and 7.37%, respectively, which are significantly improved compared with the reported values of 20% and 28% in the literature. The maximum relative error of the numerical prediction for the profile dimension appears at the edge of the printable window. Based on the numerical prediction, the effects of the process parameters on deposited filaments are also investigated. The deposition status starts from over-deposition to pressed deposition and finally transitions to freeform deposition when V* and H* increase, while on average, the velocity of ink in the nozzle has no significant effect on the deposition status or profile dimension. The deposition status should be taken into consideration for the effect of process parameters on the profile dimension: the width decreases with V* in pressed deposition and freeform deposition; the height remains the same with V* in pressed deposition but decreases with V* in freeform deposition; the width decreases with H* in pressed deposition but remains the same with H* in freeform deposition; the height increases with H* in pressed deposition but remains the same with H* in freeform deposition. The present study suggests a material-saving and effective numerical prediction method for the deposition status and profile dimension of the deposited filaments and the investigation of the effects of process parameters in DIW. It also provides a viable basis for future work on the elaboration of numerical modeling of the overall DIW 3D printing process.

## Figures and Tables

**Table 1:** Material properties of the commercially available MW-based ink Nivea Crème.

Parameter	Density *ρ* (kg/m^3^)	Surface Tension Coefficient *σ* (mN/m)	Static Contact Angle *θ* (°)	Limiting Dynamic Viscosity *μ*_0_ (Pa·s)	Yield Stress *τ*_0_ (Pa)	Consistency Index *K* (Pa·s^n^)	Flow Index *n*	Die-Swelling Factor *β*
Value	972	43	180	1.58 × 10^6^	563	867	0.045	1

**Table 2:** Experimental width under different process parameters (width unit: mm).

H*	0.3		0.6		0.9		1.2		1.5	
V*	*v_e_*/(mm/s)		*v_e_*/(mm/s)		*v_e_*/(mm/s)		*v_e_*/(mm/s)		*v_e_*/(mm/s)	
	6	9	6	9	6	9	6	9	6	9
1.2	+	+	1.00 ± 0.04	1.07 ± 0.06	0.81 ± 0.03	0.79 ± 0.03	0.80 ± 0.05	0.77 ± 0.03	0.79 ± 0.03	0.77 ± 0.03
0.8	+	+	+	+	1.10 ± 0.02	1.06 ± 0.04	0.97 ± 0.03	0.95 ± 0.04	0.97 ± 0.03	0.95 ± 0.03
0.4	+	+	+	+	+	+	+	+	1.43 ± 0.05	1.35 ± 0.04

Note: + means over-deposition status.

**Table 3:** Experimental height under different process parameters (height unit: mm).

H*	0.3		0.6		0.9		1.2		1.5	
V*	*v_e_*/(mm/s)		*v_e_*/(mm/s)		*v_e_*/(mm/s)		*v_e_*/(mm/s)		*v_e_*/(mm/s)	
	6	9	6	9	6	9	6	9	6	9
1.2	+	+	0.5 ± 0.01	0.5 ± 0.02	0.75 ± 0.01	0.75 ± 0.01	0.80 ± 0.05	0.77 ± 0.01	0.79 ± 0.03	0.77 ± 0.01
0.8	+	+	+	+	0.75 ± 0.01	0.75 ± 0.01	0.97 ± 0.03	0.95 ± 0.04	0.97 ± 0.03	0.95 ± 0.03
0.4	+	+	+	+	+	+	+	+	1.25 ± 0.02	1.25 ± 0.01

Note: + means over-deposition status.

**Table 4:** Simulated width under different process parameters (width unit: mm).

H*	0.3		0.6		0.9		1.2		1.5	
V*	*v_e_*/(mm/s)		*v_e_*/(mm/s)		*v_e_*/(mm/s)		*v_e_*/(mm/s)		*v_e_*/(mm/s)	
	6	9	6	9	6	9	6	9	6	9
1.2	+	+	1.00	1.00	0.89	0.87	0.81	0.80	0.81	0.80
0.8	+	+	+	+	1.10	1.10	0.94	0.92	0.97	0.94
0.4	+	+	+	+	+	+	+	+	1.35	1.30

Note: + means over-deposition status.

**Table 5:** Simulated height under different process parameters (height unit: mm).

H*	0.3		0.6		0.9		1.2		1.5	
V*	*v_e_*/(mm/s)		*v_e_*/(mm/s)		*v_e_*/(mm/s)		*v_e_*/(mm/s)		*v_e_*/(mm/s)	
	6	9	6	9	6	9	6	9	6	9
1.2	+	+	0.50	0.50	0.73	0.71	0.78	0.78	0.78	0.76
0.8	+	+	+	+	0.74	0.74	0.93	0.93	0.90	0.88
0.4	+	+	+	+	+	+	+	+	1.25	1.23

Note: + means over-deposition status.

**Table 6:** Relative error of experimental and simulated width under different process parameters.

H*	0.3		0.6		0.9		1.2		1.5	
V*	*v_e_*/(mm/s)		*v_e_*/(mm/s)		*v_e_*/(mm/s)		*v_e_*/(mm/s)		*v_e_*/(mm/s)	
	6	9	6	9	6	9	6	9	6	9
1.2	+	+	0	6.54%	9.88%	10.13%	1.25%	3.90%	2.53%	4.00%
0.8	+	+	+	+	0	3.77%	4.44%	3.16%	0	1.05%
0.4	+	+	+	+	+	+	+	+	5.59%	3.70%

Note: + means over-deposition status.

**Table 7:** Relative error of experimental and simulated height under different process parameters.

H*	0.3		0.6		0.9		1.2		1.5	
V*	*v_e_*/(mm/s)		*v_e_*/(mm/s)		*v_e_*/(mm/s)		*v_e_*/(mm/s)		*v_e_*/(mm/s)	
	6	9	6	9	6	9	6	9	6	9
1.2	+	+	0	0	2.67%	5.33%	2.50%	1.30%	1.27%	1.30%
0.8	+	+	+	+	1.33%	1.33%	4.12%	2.10%	7.22%	7.37%
0.4	+	+	+	+	+	+	+	+	0	1.60%

Note: + means over-deposition status.

## Data Availability

Data will be made available upon request.

## Supplementary Materials

The following supporting information can be downloaded at https://www.mdpi.com/article/10.3390/polym17050573/s1: Video S1: Simulated over deposition; Video S2: Simulated pressed deposition; Video S3: Simulated freeform deposition.

## Nomenclature

DIW	Direct ink writing
MEAM	Material extrusion additive manufacturing
CFD	Computational fluid dynamics
PLA	Poly-lactic acid
VOF	Volume of fluid
MW	Microcrystalline wax
α	Phase fraction
$V_{i}$	Volume of ink in a mesh
$V_{m}$	Total volume of a mesh
$\rho_{s}$	Density of single continuum
$\mu_{s}$	Viscosity of single continuum
ρ	Density of ink
$\rho_{a}$	Density of air
μ	Viscosity of ink
$\mu_{a}$	Viscosity of air
$\dot{\gamma }$	Shear rate
$\mu_{0}$	Limiting dynamic viscosity
$\tau_{0}$	Yield stress
K	Consistency index
n	Flow index
U	Velocity field
p	Pressure
g	Gravitational acceleration vector
$F_{\sigma }$	Surface tension
σ	Surface tension coefficient
κ	Surface curvature
n	Normal vector to ink surface
$\hat{n}$	Unit vector normal to ink surface
${\hat{n}}_{w}$	Unit vector normal to wall
${\hat{t}}_{w}$	Unit vector tangent to wall
θ	Static contact angle
$U_{r}$	Velocity vector compressing two-phase free surface
c	Controllable compression factor
$v_{e}$	Average velocity in nozzle
$D_{p}$	Piston diameter
$D_{n}$	Outer diameter of nozzle
$d_{n}$	Inner diameter of nozzle
$v_{p}$	Piston velocity
$L_{n}$	Nozzle length
h	Distance between nozzle bottom and substrate
$v_{n}$	Nozzle velocity
$V^{*}$	$\mathrm{Dimensionless}\;\mathrm{nozzle}\;\mathrm{velocity},=v_{n}/v_{e}$
$H^{*}$	$\mathrm{Dimensionless}\;\mathrm{height},=h/\beta d_{n}$
β	Die-swelling factor
γ	$\mathrm{Ratio}\;\mathrm{of}\;D_{n}$ $\mathrm{to}\;d_{n}$ $,=D_{n}/d_{n}$
ε	$=\left(\theta -\frac{\sin2\theta }{2}\right){\left(\frac{1}{1-\cos\theta }\right)}^{2}$
